# Di-μ-chlorido-bis­[(2-amino­benzamide-κ^2^ *N* ^2^,*O*)chlorido­copper(II)]

**DOI:** 10.1107/S1600536813021879

**Published:** 2013-08-10

**Authors:** Maamar Damous, George Dénès, Sofiane Bouacida, Meriem Hamlaoui, Hocine Merazig, Jean-Claude Daran

**Affiliations:** a Unité de Recherche de Chimie de l’Environnement et Moléculaire Structurale, CHEMS, Université Constantine 1, 25000 , Algeria; b Laboratory of Solid State Chemistry and Mössbauer Spectroscopy, Laboratories for Inorganic Materials, Department of Chemistry and Biochemistry, Concordia University, Montreal, Quebec, H3G 1M8, Canada; c Département Sciences de la Matière, Faculté des Sciences Exactes et Sciences de la Nature et de la Vie, Université Oum El Bouaghi 04000, Algeria; d Laboratoire de Chimie de Coordination, UPR CNRS 8241, 205 route de Narbonne, 31077 Toulouse cedex, France

## Abstract

The title compound, [Cu_2_Cl_4_(C_7_H_8_N_2_O)_2_], crystallizes as discrete [Cu*L*Cl_2_]_2_ (*L* = 2-amino­benzamide) dimers with inversion symmetry. Each Cu^II^ ion is five-coordinated and is bound to two bridging chloride ligands, a terminal chloride ligand and a bidentate 2-amino­benzamide ligand. The crystal structure exhibits alternating layers parallel to (010) along the *b-*axis direction. In the crystal, the components are linked *via* N—H⋯Cl hydrogen bonds, forming a three-dimensional network. These inter­actions link the mol­ecules within the layers and also link the layers together and reinforce the cohesion of the structure.

## Related literature

For general background to 2-amino­benzamide derivatives, see: Nagaoka *et al.* (2006 ▶); Butsch *et al.* (2011 ▶); Kapoor *et al.* (2010 ▶). For related structures, see: Yang *et al.* (2012 ▶); Lah *et al.* (2006 ▶). For standard bond lengths, see: Allen (2002 ▶)
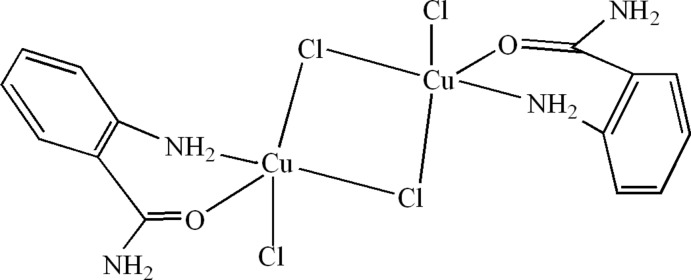


## Experimental

### 

#### Crystal data

[Cu_2_Cl_4_(C_7_H_8_N_2_O)_2_]
*M* *_r_* = 541.21
Monoclinic, 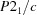

*a* = 8.1888 (5) Å
*b* = 13.8545 (6) Å
*c* = 8.1592 (4) Å
β = 98.771 (5)°
*V* = 914.85 (8) Å^3^
*Z* = 2
Mo *K*α radiation
μ = 2.93 mm^−1^
*T* = 180 K
0.15 × 0.13 × 0.12 mm

#### Data collection

Agilent Xcalibur (Sapphire1) diffractometer
Absorption correction: multi-scan (*CrysAlis PRO*; Agilent, 2011 ▶) *T*_min_ = 0.699, *T*_max_ = 1
5578 measured reflections
2058 independent reflections
1897 reflections with *I* > 2σ(*I*)
*R*_int_ = 0.022

#### Refinement

*R*[*F* ^2^ > 2σ(*F* ^2^)] = 0.022
*wR*(*F* ^2^) = 0.058
*S* = 1.04
2058 reflections
118 parameters
H-atom parameters constrained
Δρ_max_ = 0.41 e Å^−3^
Δρ_min_ = −0.41 e Å^−3^

### 

Data collection: *CrysAlis PRO* (Agilent, 2011 ▶); cell refinement: *CrysAlis PRO*; data reduction: *CrysAlis PRO*; program(s) used to solve structure: *SIR2002* (Burla *et al.*, 2003 ▶); program(s) used to refine structure: *SHELXL97* (Sheldrick, 2008 ▶); molecular graphics: *ORTEP-3 for Windows* (Farrugia, 2012 ▶) and *DIAMOND* (Brandenburg & Berndt, 2001 ▶); software used to prepare material for publication: *WinGX* (Farrugia, 2012 ▶).

## Supplementary Material

Crystal structure: contains datablock(s) I. DOI: 10.1107/S1600536813021879/hg5337sup1.cif

Structure factors: contains datablock(s) I. DOI: 10.1107/S1600536813021879/hg5337Isup2.hkl

Additional supplementary materials:  crystallographic information; 3D view; checkCIF report

## Figures and Tables

**Table 1:** Hydrogen-bond geometry (Å, °)

*D*—H⋯*A*	*D*—H	H⋯*A*	*D*⋯*A*	*D*—H⋯*A*
N1—H1*A*⋯Cl2^i^	0.9200	2.4100	3.3113 (16)	166.00
N2—H2*A*⋯Cl1^ii^	0.8800	2.7800	3.6439 (16)	169.00
N2—H2*B*⋯Cl2^iii^	0.8800	2.5400	3.3493 (17)	153.00

Symmetry codes: (i) 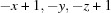; (ii) 
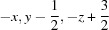
; (iii) 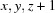.

**Fig. 1.**
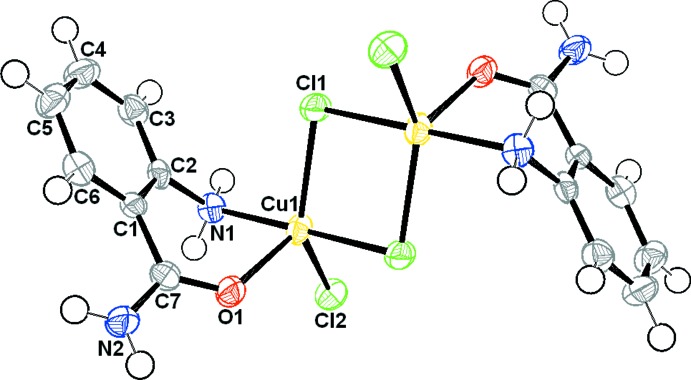
(Farrugia, 2012) The molecule structure of the title dimer with the atomic labelling scheme·Displacement are drawn at the 50% probability level. H atoms are represented as small spheres of arbitrary radius. Only the contents of the asymmetric unit are numbered.

**Fig. 2.**
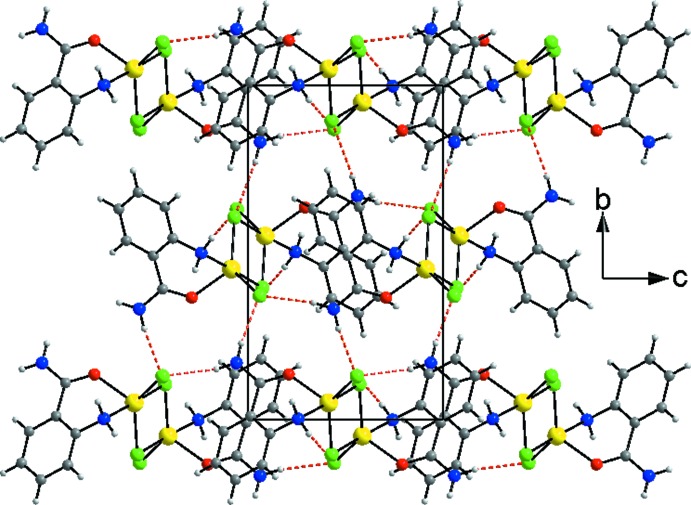
(Brandenburg & Berndt, 2001) A diagram of the layered crystal packing in (I), viewed down the b axis, showing layers parallel to (010) and hydrogen bond connections as dashed line.

## supplementary crystallographic information

### 1. Comment

2-aminobenzamide derivatives are well known compounds as anticancer agents (Nagaoka *et al.*, 2006). In addition, it was reported that some 2-aminobenzamide derivatives possessed biological activities, such as Anti-herpes simplex virus activity (Yang *et al.*, 2012). As part of our ongoing studies of complexes based on copper and derivates we report here synthesis and the crystal structure of the title compound, obtained by the reaction of 2-aminobenzamide ligand with copper(II) chloride. The molecular structure of (I), and the atomic numbering used, is illustrated in Fig. 1. The asymmetric unit of (I) consists of one-half of the molecule, with the other half generated by a crystallographic inversion center. All bond distances and angles are within the ranges of accepted values(CSD, Allen, 2002) The complex contain five-coordinate Cu atoms (Fig. 1) with may be described either as square pyramidal with C11 apically bound to a pseudoplanar Cu1—O1—C12—Cl1a—N1 (a:-*x*, -*y*,1 - *z*) fragment or as trigonal bipyramidal with N1 and Cl1a apical (Butsch *et al.*, 2011; Kapoor *et al.*, 2010). The Cu atoms are linked by double Cl atoms bridges, resulting in the formation of dimer. The two Cu atoms, separated by 3.430 (1) Å, are doubly bridged by two chlorido ligands. The bridge is far from symmetrical with Cu—Cl1 and Cu—Cl1a (with a: -*x*, -*y*,1 - *z*) distances of 2.3983 (5) and 2.2990 (6) Å, respectively, and a Cu—Cl1-Cua bridging angle of 93.77 (2)°. The 2-aminobenzamide ligand binds to a single Cu metal ion within the dimer in a chelating manner [Cu—N1: 1.9923 (15) Å and Cu–O1: 2.0988 (13) Å]. The fifth coordination site is occupied by a terminal chlorido ligand at a distance of 2.3043 (6) Å (Lah *et al.*, 2006).

The crystal structure exhibit alternating layers parallel to (010) plane along the *b* axis (Fig. 2). In the crystal, the components of the structure are linked *via* intermolecular N—H···Cl hydrogen bonds to form a three-dimensional network (Table1 and Fig.2) These interaction bonds link the molecules within the layers and also link the layers together and reinforcing the cohesion of the structure.

### 2. Experimental

An aqueous acidic solution of copper(II) chloride was added to an aqueous solution of the 2-aminobenzamide ligand (*L*) (1:l mol ratio). The mixture was then stirred for several hours during which time darkish green crystals of [CuLCl_2_]_2_ were deposited. This crystalline product was collected and washed with ether and was carefully isolated under polarizing microscope for analysis by X-ray diffraction.

### 3. Refinement

All non-H atoms were refined with anisotropic atomic displacement parameters. The remaining H atoms were localized on Fourier maps but introduced in calculated positions and treated as riding on their parent atoms (C and N) with C—H=0.95 Å and N—H=0.88 or 0.92 Å and *U*_iso_(H)=1.2*U*_eq_(C or N).

### Crystal data

**Table:**

[Cu_2_Cl_4_(C_7_H_8_N_2_O)_2_]	*F*(000) = 540
*M**_r_* = 541.21	*D*_x_ = 1.965 Mg m^−^^3^
Monoclinic, *P*2_1_/*c*	Mo *K*α radiation, λ = 0.71073 Å
Hall symbol: -P 2ybc	Cell parameters from 3363 reflections
*a* = 8.1888 (5) Å	θ = 2.9–28.2°
*b* = 13.8545 (6) Å	µ = 2.93 mm^−^^1^
*c* = 8.1592 (4) Å	*T* = 180 K
β = 98.771 (5)°	Cube, green
*V* = 914.85 (8) Å^3^	0.15 × 0.13 × 0.12 mm
*Z* = 2	

### Data collection

**Table:**

Agilent Xcalibur (Sapphire1) diffractometer	2058 independent reflections
Radiation source: fine-focus sealed tube	1897 reflections with *I* > 2σ(*I*)
Graphite monochromator	*R*_int_ = 0.022
Detector resolution: 8.2632 pixels mm^-1^	θ_max_ = 28.3°, θ_min_ = 2.9°
ω scans	*h* = −9→10
Absorption correction: multi-scan (*CrysAlis PRO*; Agilent, 2011)	*k* = −17→18
*T*_min_ = 0.699, *T*_max_ = 1	*l* = −10→10
5578 measured reflections	

### Refinement

**Table:**

Refinement on *F*^2^	Primary atom site location: structure-invariant direct methods
Least-squares matrix: full	Secondary atom site location: difference Fourier map
*R*[*F*^2^ > 2σ(*F*^2^)] = 0.022	Hydrogen site location: inferred from neighbouring sites
*wR*(*F*^2^) = 0.058	H-atom parameters constrained
*S* = 1.04	*w* = 1/[σ^2^(*F*_o_^2^) + (0.0307*P*)^2^ + 0.3739*P*] where *P* = (*F*_o_^2^ + 2*F*_c_^2^)/3
2058 reflections	(Δ/σ)_max_ = 0.003
118 parameters	Δρ_max_ = 0.41 e Å^−^^3^
0 restraints	Δρ_min_ = −0.41 e Å^−^^3^

### Special details

**Table:**

Experimental. Absorption correction: empirical using spherical harmonics, implemented in SCALE3 ABSPACK scaling algorithm CrysAlis PRO (Agilent, 2011).
Geometry. Bond distances, angles *etc*. have been calculated using the rounded fractional coordinates. All su's are estimated from the variances of the (full) variance-covariance matrix. The cell e.s.d.'s are taken into account in the estimation of distances, angles and torsion angles
Refinement. Refinement of *F*^2^ against ALL reflections. The weighted *R*-factor *wR* and goodness of fit *S* are based on *F*^2^, conventional *R*-factors *R* are based on *F*, with *F* set to zero for negative *F*^2^. The threshold expression of *F*^2^ > σ(*F*^2^) is used only for calculating *R*-factors(gt) *etc*. and is not relevant to the choice of reflections for refinement. *R*-factors based on *F*^2^ are statistically about twice as large as those based on *F*, and *R*- factors based on ALL data will be even larger.

### Fractional atomic coordinates and isotropic or equivalent isotropic displacement parameters (Å^2^)

**Table:**

	*x*	*y*	*z*	*U*_iso_*/*U*_eq_	
Cu1	0.18491 (3)	−0.04957 (2)	0.58700 (3)	0.0126 (1)	
Cl1	0.05270 (6)	0.10533 (3)	0.57135 (6)	0.0161 (1)	
Cl2	0.36324 (6)	−0.12970 (3)	0.44121 (6)	0.0162 (1)	
O1	0.15024 (17)	−0.13480 (9)	0.79155 (16)	0.0156 (4)	
N1	0.37924 (19)	−0.00155 (11)	0.74251 (18)	0.0126 (4)	
N2	0.1556 (2)	−0.16908 (11)	1.0607 (2)	0.0180 (5)	
C1	0.2398 (2)	−0.00937 (12)	0.9884 (2)	0.0107 (5)	
C2	0.3341 (2)	0.04226 (12)	0.8882 (2)	0.0111 (5)	
C3	0.3819 (2)	0.13689 (13)	0.9288 (2)	0.0147 (5)	
C4	0.3337 (3)	0.18053 (13)	1.0665 (2)	0.0181 (5)	
C5	0.2408 (3)	0.13070 (13)	1.1664 (2)	0.0171 (5)	
C6	0.1958 (2)	0.03556 (13)	1.1275 (2)	0.0140 (5)	
C7	0.1801 (2)	−0.10930 (12)	0.9406 (2)	0.0122 (5)	
H1A	0.43529	0.04286	0.68835	0.0152*	
H1B	0.44964	−0.05231	0.77350	0.0152*	
H2A	0.11781	−0.22765	1.03652	0.0216*	
H2B	0.17697	−0.15031	1.16476	0.0216*	
H3	0.44744	0.17143	0.86209	0.0176*	
H4	0.36477	0.24546	1.09246	0.0217*	
H5	0.20810	0.16101	1.26070	0.0206*	
H6	0.13389	0.00073	1.19728	0.0168*	

### Atomic displacement parameters (Å^2^)

**Table:**

	*U*^11^	*U*^22^	*U*^33^	*U*^12^	*U*^13^	*U*^23^
Cu1	0.0105 (1)	0.0152 (1)	0.0119 (1)	0.0003 (1)	0.0005 (1)	−0.0027 (1)
Cl1	0.0141 (2)	0.0125 (2)	0.0198 (2)	0.0006 (2)	−0.0036 (2)	−0.0023 (2)
Cl2	0.0160 (2)	0.0175 (2)	0.0162 (2)	0.0013 (2)	0.0059 (2)	−0.0026 (2)
O1	0.0214 (7)	0.0129 (6)	0.0121 (6)	−0.0036 (5)	0.0016 (5)	−0.0002 (5)
N1	0.0118 (7)	0.0148 (7)	0.0119 (7)	−0.0002 (6)	0.0038 (6)	0.0005 (6)
N2	0.0280 (10)	0.0118 (7)	0.0140 (8)	−0.0036 (7)	0.0026 (7)	−0.0001 (6)
C1	0.0100 (8)	0.0100 (8)	0.0113 (8)	0.0013 (7)	−0.0007 (7)	0.0004 (7)
C2	0.0084 (8)	0.0147 (8)	0.0094 (8)	0.0021 (7)	−0.0011 (7)	0.0005 (7)
C3	0.0141 (9)	0.0149 (8)	0.0146 (9)	−0.0020 (7)	0.0009 (7)	0.0026 (7)
C4	0.0193 (10)	0.0124 (8)	0.0217 (10)	−0.0016 (7)	0.0004 (8)	−0.0032 (7)
C5	0.0177 (10)	0.0176 (9)	0.0162 (9)	0.0016 (8)	0.0030 (8)	−0.0049 (7)
C6	0.0118 (9)	0.0167 (9)	0.0135 (9)	0.0006 (7)	0.0022 (7)	0.0026 (7)
C7	0.0103 (9)	0.0127 (8)	0.0135 (9)	0.0016 (7)	0.0018 (7)	0.0014 (7)

### Geometric parameters (Å, º)

**Table:**

Cu1—Cl1	2.3983 (5)	C1—C2	1.403 (2)
Cu1—Cl2	2.3043 (6)	C1—C6	1.389 (2)
Cu1—O1	2.0988 (13)	C1—C7	1.500 (2)
Cu1—N1	1.9923 (15)	C2—C3	1.394 (2)
Cu1—Cl1^i^	2.2990 (6)	C3—C4	1.385 (2)
O1—C7	1.254 (2)	C4—C5	1.382 (3)
N1—C2	1.433 (2)	C5—C6	1.392 (3)
N2—C7	1.322 (2)	C3—H3	0.9500
N1—H1A	0.9200	C4—H4	0.9500
N1—H1B	0.9200	C5—H5	0.9500
N2—H2A	0.8800	C6—H6	0.9500
N2—H2B	0.8800		
			
Cl1—Cu1—Cl2	136.06 (2)	C2—C1—C6	118.81 (15)
Cl1—Cu1—O1	115.54 (4)	C2—C1—C7	120.37 (14)
Cl1—Cu1—N1	92.60 (5)	C6—C1—C7	120.74 (15)
Cl1—Cu1—Cl1^i^	86.23 (2)	N1—C2—C1	120.14 (15)
Cl2—Cu1—O1	108.22 (4)	N1—C2—C3	119.81 (15)
Cl2—Cu1—N1	88.98 (5)	C1—C2—C3	120.04 (15)
Cl1^i^—Cu1—Cl2	95.55 (2)	C2—C3—C4	119.93 (16)
O1—Cu1—N1	82.72 (6)	C3—C4—C5	120.73 (17)
Cl1^i^—Cu1—O1	93.00 (4)	C4—C5—C6	119.28 (16)
Cl1^i^—Cu1—N1	174.57 (5)	C1—C6—C5	121.19 (16)
Cu1—Cl1—Cu1^i^	93.77 (2)	O1—C7—C1	121.32 (15)
Cu1—O1—C7	125.63 (11)	N2—C7—C1	117.78 (15)
Cu1—N1—C2	112.82 (11)	O1—C7—N2	120.88 (16)
C2—N1—H1A	109.00	C2—C3—H3	120.00
C2—N1—H1B	109.00	C4—C3—H3	120.00
H1A—N1—H1B	108.00	C3—C4—H4	120.00
Cu1—N1—H1A	109.00	C5—C4—H4	120.00
Cu1—N1—H1B	109.00	C4—C5—H5	120.00
H2A—N2—H2B	120.00	C6—C5—H5	120.00
C7—N2—H2A	120.00	C1—C6—H6	119.00
C7—N2—H2B	120.00	C5—C6—H6	119.00
			
Cl2—Cu1—Cl1—Cu1^i^	94.09 (3)	Cu1—N1—C2—C1	55.55 (18)
O1—Cu1—Cl1—Cu1^i^	−91.53 (5)	C6—C1—C2—N1	−179.03 (15)
N1—Cu1—Cl1—Cu1^i^	−174.69 (5)	C7—C1—C6—C5	−175.43 (17)
Cl1^i^—Cu1—Cl1—Cu1^i^	0.00 (4)	C2—C1—C7—O1	−29.6 (2)
Cl1—Cu1—Cl1^i^—Cu1^i^	0.00 (4)	C6—C1—C2—C3	0.0 (2)
Cl2—Cu1—Cl1^i^—Cu1^i^	−135.94 (2)	C6—C1—C7—O1	147.06 (17)
O1—Cu1—Cl1^i^—Cu1^i^	115.41 (4)	C6—C1—C7—N2	−31.2 (2)
Cl1—Cu1—O1—C7	−53.92 (15)	C2—C1—C7—N2	152.19 (16)
Cl2—Cu1—O1—C7	121.99 (14)	C2—C1—C6—C5	1.3 (3)
N1—Cu1—O1—C7	35.49 (15)	C7—C1—C2—C3	176.68 (15)
Cl1^i^—Cu1—O1—C7	−141.17 (14)	C7—C1—C2—N1	−2.3 (2)
Cl1—Cu1—N1—C2	55.31 (11)	C1—C2—C3—C4	−1.2 (3)
Cl2—Cu1—N1—C2	−168.63 (11)	N1—C2—C3—C4	177.83 (17)
O1—Cu1—N1—C2	−60.11 (11)	C2—C3—C4—C5	1.2 (3)
Cu1—O1—C7—C1	2.6 (2)	C3—C4—C5—C6	0.1 (3)
Cu1—O1—C7—N2	−179.23 (12)	C4—C5—C6—C1	−1.3 (3)
Cu1—N1—C2—C3	−123.45 (14)		

Symmetry code: (i) −*x*, −*y*, −*z*+1.

### Hydrogen-bond geometry (Å, º)

**Table:**

*D*—H···*A*	*D*—H	H···*A*	*D*···*A*	*D*—H···*A*
N1—H1*A*···Cl2^ii^	0.9200	2.4100	3.3113 (16)	166.00
N2—H2*A*···Cl1^iii^	0.8800	2.7800	3.6439 (16)	169.00
N2—H2*B*···Cl2^iv^	0.8800	2.5400	3.3493 (17)	153.00

Symmetry codes: (ii) −*x*+1, −*y*, −*z*+1; (iii) −*x*, *y*−1/2, −*z*+3/2; (iv) *x*, *y*, *z*+1.
